# Robust Bayesian hypothesis testing with the hierarchical EZ-DDM

**DOI:** 10.3758/s13428-026-03066-1

**Published:** 2026-05-28

**Authors:** Adriana F. Chávez De la Peña, Eunice Shin, Joachim Vandekerckhove

**Affiliations:** 1 Department of Cognitive Sciences, University of California, Irvine, Irvine, CA 92697-5100 USA; 2 Department of Statistics, University of California, Irvine, Irvine, CA 92697-5100 USA; 3 Department of Logic & Philosophy of Science, University of California, Irvine, CA 92697-5100 USA

**Keywords:** Robust inference, Cognitive psychometrics, EZ diffusion, Hypothesis testing, Hierarchical Bayesian modeling

## Abstract

The EZ-diffusion model (EZ-DDM) uses a method of moments to provide closed-form estimators for the three-parameter drift-diffusion model from summary statistics. In previous work, we showed that using the sampling distributions of these statistics enables the implementation of hierarchical EZ-DDM extensions, supporting scalable Bayesian inference in cognitive psychometrics applications. However, the summary statistics used in EZ-DDM implementations (the mean and variance of the response time distribution) are sensitive to contaminant data points, limiting its utility in real-world applications. To address this, we propose a variation on the EZ-DDM implementation in which the summary statistics are replaced with robust alternatives, substituting mean RT with median RT and RT variance with an estimate derived from the interquartile range. We explore and evaluate the effectiveness of this substitution through simulation studies using a within-subject *t* test design across varying sample sizes and effect sizes. We show that the robust variant matched the diagnostic accuracy of the EZ-DDM implementation on uncontaminated data while maintaining diagnostic accuracy under contamination, unlike the standard model. This extension preserves efficiency while adding robustness in real-world applications. We recommend using the robust EZ-DDM in practical applications.

Robustness is a necessary quality for trustworthy scientific inference. Broadly defined, it refers to the capacity of a method to preserve its intended function in the presence of perturbations that threaten its integrity. We want to ensure that the methods we use do not break down in the presence of small departures from the assumptions underlying their theoretical foundations (Lee et al., 2019). Such robust procedures allow valid inference even in the presence of skewness, contaminant data points, heteroscedasticity, and other irregularities commonly encountered in real-world data sets (Huber & Ronchetti, 2009).

Estimation methods that rely on summary statistics that are sensitive to contaminants and other irregularities in the data are not *robust*. This is the case for the EZ-DDM (Wagenmakers et al., 2007), a simplification of the drift diffusion model (Ratcliff, 1978) that enables parameter estimation for the drift rate, boundary separation, and non-decision time parameters from three summary statistics: the mean and variance of the RT distribution, and the accuracy rate. In this paper, we explore a robust implementation of the EZ-DDM, in which the mean and variance are replaced by two approximations that are less sensitive to contaminants: the median and variance estimate derived from the interquartile range (IQR). Specifically, we embed the proposed modification into the hierarchical Bayesian framework that we introduced in previous work (Chávez De la Peña & Vandekerckhove, 2026) and evaluate its performance in simulation studies.

## A hierarchical Bayesian EZ-DDM

In previous work, we introduced a Bayesian hierarchical EZ-DDM implementation that recasts the observed accuracy rate as the binomial rate for correct response counts and uses the sampling distributions of the mean and variance of RT to define a proxy likelihood that can be easily implemented in probabilistic programming languages (Chávez De la Peña & Vandekerckhove, 2026). This proxy enables latent-variable and covariate regression structures and is well suited for scalable inference due to its reliance on summary statistics.

## A robust Bayesian EZ-DDM

The robust implementation of the EZ-DDM substitutes the mean and variance of the RT distribution with the sample median and the following variance estimate:

1 $$\begin{aligned} \widehat{\text {Var}}_{\text {IQR}} = {\left( \frac{Q_{3} - Q_{1}}{1.349}\right) ^2} \end{aligned}$$

where $Q_{1}$ and $Q_{3}$ are the first and third quartiles of the RT distribution.

Aside from the summary statistic substitution, nothing in the model changes – the robust implementation of the hierarchical Bayesian EZ-DDM continues to use the sampling distributions of the mean and variance of RTs, thus preserving the same proxy likelihood structure.

## Hypothesis testing simulation design

We evaluated and compared the performance of the robust and standard implementations of the hierarchical Bayesian EZ-DDM for hypothesis testing through simulation studies. We generated trial-level data from a Wiener diffusion process with a within-subject *t* test design on the drift rate parameter $\nu _{p,k}$ given by the following hierarchical meta-regression model:

2 $$\begin{aligned} \nu ^\text {pred}_{p,k}&= \mu _\nu + \beta X_k \\ \nu _{p,k}&\sim \mathcal {N}\!\left( \nu ^\text {pred}_{p,k},\ \sigma ^2_\nu \right) \nonumber \\ \alpha _{p}&\sim \mathcal {N}\!\left( \mu _\alpha ,\ \sigma ^2_\alpha \right) \nonumber \\ \tau _{p}&\sim \mathcal {N}\!\left( \mu _\tau ,\ \sigma ^2_\tau \right) \nonumber \end{aligned}$$

such that the predicted drift rate for participant *p* in condition *k* ($\nu ^\text {pred}_{p,k}$) is defined as the linear combination of a population-level drift rate intercept $\mu _\nu$ and the population-level regression coefficient capturing the condition effect $\beta$ (with $X_k \in \{0,1\}$ as the condition indicator). In this data-generating model, the dispersion of the person-by-condition drift rates $\nu _{p,k}$ around these predicted values is captured by the variance parameter $\sigma ^2_\nu$, while hierarchical parameters $\mu _\alpha$, $\mu _\tau$, and $\sigma ^2_\alpha$, $\sigma ^2_\tau$ capture the mean and variance of the population-level distributions of the boundary separation $\alpha _p$ and non-decision time $\tau _p$ parameters, respectively.

In our simulation studies, we generated 1000 independent data sets for each design cell defined by (1) number of participants *P*, (2) number of trials per condition *T*, and (3) true fixed effect size $\beta$. Across all data sets generated, we fixed the value of all variance parameters $\sigma ^2_\nu = 0.75, \sigma ^2_\alpha = 0.5, \sigma ^2_\tau = 0.1$. The rest of the true parameters were generated by drawing population-level values for $\mu _\nu$, $\mu _\alpha$, and $\mu _\tau$ from uniform distributions (see below), and then sampling individual-level boundary separation ($\alpha _p$) and non-decision time ($\tau _p$) parameters from the corresponding group-level normal distributions. Similarly, we drew participant-specific drift rates $\nu _{p,k}$ from the regression structure described in Eq. 2.

### Comparison conditions

Each simulated dataset consisted of clean trial-level data from a Wiener diffusion process. For each dataset, we constructed a contaminated counterpart by replacing 5% of each participant’s trials with contaminant data (distributed equally over conditions), while leaving the remaining 95% of trials identical to the clean data. From both versions (clean and contaminated), we then computed standard and robust summary statistics, yielding a $2 \times 2$ factorial comparison design: *data integrity* (clean vs. contaminated) $\times$ *summary method* (standard vs. robust).

### Contamination procedure

In each participant-by-condition run of trials, we contaminated 5% of trials using a two-stage procedure. Following (Vandekerckhove et al., 2008), we generated a binary indicator for each of these trials to determine the type of contamination. With 50% probability, the trial was a *delayed startup*, for which we added uniform noise between 2s and 3s to the observed RT. For the other contaminant trials, we generated a *guess* trial by setting the drift rate to 0 and redrawing the observation.

**Fig. 1:**
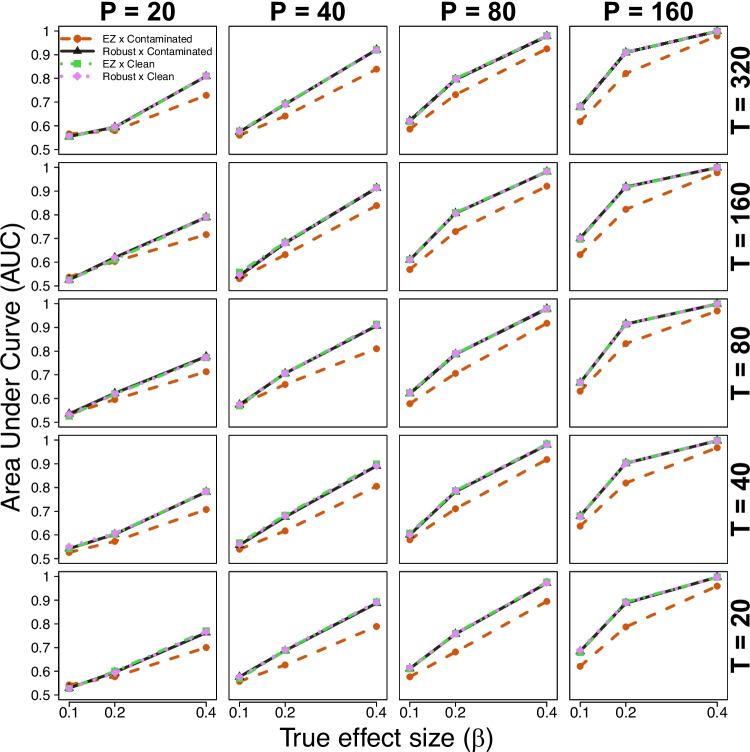
Simulation study results (diagnostic accuracy). Each panel shows the AUC (*vertical axis*) for different true effect sizes (*horizontal axis*) and simulation comparison conditions (different lines), across different trial and participant sizes (*rows and columns*)

## Simulation study

We conducted an extensive simulation study in which we generated 1000 datasets for each cell defined by a $4 \times 5 \times 4$ factorial design. This design included four levels for the number of participants $P \in \{20, 40, 80, 160\}$, five levels for the number of trials per condition $T \in \{20, 40, 80, 160, 320\}$, and four levels for the true fixed effect size $\beta \in \{0.0, 0.1, 0.2, 0.4\}$. Across all datasets, the true values for all variance parameters were fixed at $\sigma ^2_\nu = 0.75, \sigma ^2_\alpha = 0.5, \sigma ^2_\tau = 0.1$. Each dataset was generated from its own set of true values for $\mu _\nu$, $\mu _\alpha$, and $\mu _\tau$ sampled from the following uniform distributions:

$$\begin{aligned} \mu _\nu&\sim \mathcal {U}\!\left( -3, 3\right) \nonumber&\mu _\alpha&\sim \mathcal {U}\!\left( 2, 4\right) \nonumber&\mu _\tau&\sim \mathcal {U}\!\left( 0.2, 0.4\right) \nonumber \end{aligned}$$

These hierarchical parameters were then used to sample individual-level boundary separation ($\alpha _p$) and non-decision time ($\tau _p$) parameters and participant-by-condition specific drift rates $\nu _{p,k}$ according to the regression structure described in Eq. 2.

The results of our simulation study focus on the effect size parameter $\beta$. First, we evaluate the diagnostic accuracy performance of the robust and standard EZ-DDM implementations under clean and contaminated data, by testing the null hypothesis $\beta = 0$ in a Bayesian framework. Then, we evaluate the parameter recovery of the effect size parameter $\beta$ across all simulation conditions.

**Fig. 2:**
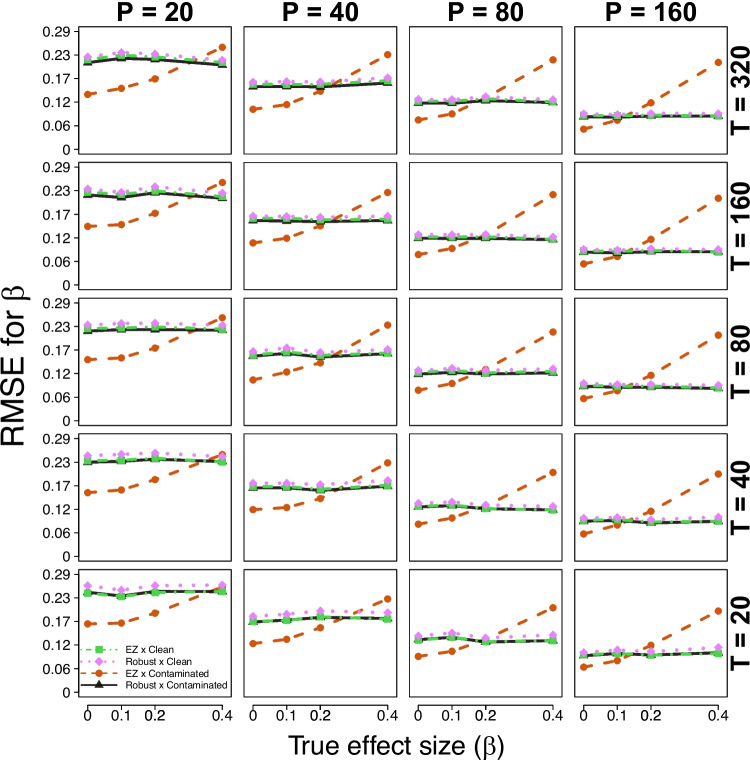
Simulation study results (parameter recovery). Each panel shows the RMSE (*vertical axis*) of the estimated effect size parameter $\beta$ (*true value on the horizontal axis*) for each comparison condition (different lines), across different trial and participant sizes (*rows and columns*)

### Diagnostic accuracy results

The hypothesis testing performance of both hierarchical Bayesian implementations was evaluated using their receiver operating characteristic (ROC) curves. To draw an ROC curve, we plot the detection rate (proportion of $\beta \ne 0$ simulations in which we correctly detect an effect) on the vertical axis and the false-alarm rate (the proportion of $\beta = 0$ simulations in which we incorrectly claim there is an effect) on the horizontal axis. The curve is then drawn by varying the decision threshold from maximally conservative (where both are 0) to maximally liberal (where both are 1). The area under the curve (AUC) metric then quantifies diagnostic accuracy, with values closer to 1 indicating stronger discrimination between true effects ($\beta \ne 0$) and null effects ($\beta = 0$).

Figure 1 summarizes the results. Within each panel, the horizontal axis displays the true effect sizes ($\beta$) and the vertical axis shows the AUC computed for each simulation condition. By definition, the AUC is 0.5 where $\beta = 0$. Different lines contrast the standard and robust EZ-DDM implementations under clean and contaminated data. Panels are arranged in a grid showing the different trial sizes per condition (*T*) across columns and the sample sizes (*P*) across rows.

The result of interest is captured by the fact that the red dashed line is dominated by all other lines, which overlap. That is, the robust and standard EZ-DDM produced nearly identical AUC results under clean data, but the standard EZ-DDM exhibits noticeable reductions in AUC under contaminated data.

### Estimation performance results

We also explore the parameter recovery performance of the robust and standard implementations of the EZ-DDM within a hierarchical Bayesian framework by computing the root mean square error (RMSE) of the estimated effect size parameter $\beta$ for each simulation condition. Our focus on $\beta$ is motivated by the fact that it is the central parameter of interest in hypothesis testing and the main determinant of the AUC diagnostic accuracy reported in Fig. 1. The RMSE summarizes overall estimation error by combining both bias and variability, with values closer to 0 indicating more accurate recovery of the true parameter.

Figure 2 summarizes the results. Within each panel, the RMSE of the estimated effect size parameter $\beta$ is plotted as a function of the true $\beta$ value, with separate lines for each of the four comparison conditions. The result of interest is that the RMSE obtained for the robust implementation across both data conditions (clean and contaminated) is comparable to that of the standard implementation under clean data, with similar RMSE values reported across all true effect sizes $\beta$. However, the RMSE of the standard implementation under contaminated data exhibits noticeable changes as a function of the true effect size $\beta$. In particular, the RMSE is lower for smaller true effect sizes $\beta$ and increases as the true effect size $\beta$ increases.

**Fig. 3:**
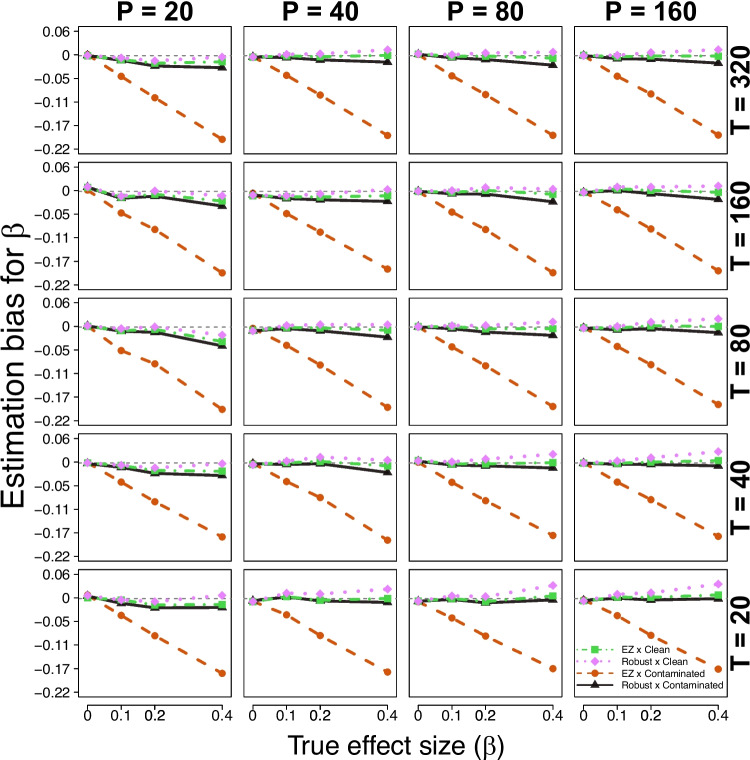
Simulation study results (parameter recovery). Each panel shows the RMSE (*vertical axis*) of the estimated effect size parameter $\beta$ (*true value on the horizontal axis*) for each comparison condition (different lines), across different trial and participant sizes (*rows and columns*)

To get a better understanding of the systematic differences in RMSE observed across simulation conditions, we inspect the estimation bias and variance components separately. Figure 3 shows the estimation bias for the effect size parameter $\beta$ as a function of its true value, across all simulation conditions. The estimation bias refers to the difference between the true and the estimated value – in this case, the mean posterior value –as such values closer to 0 indicate a more accurate (i.e., unbiased) parameter recovery. According to Fig. 3, the estimation bias for the effect size parameter $\beta$ is generally close to 0 across most simulation conditions. However, under the standard implementation of the hierarchical EZ-DDM under contaminated data, the effect size parameter seems to be systematically underestimated for $\beta \ne 0$, a result that increases notably as the true effect size $\beta$ increases.

To further investigate the change in performance observed in the standard implementation under contaminated data, we conducted additional simulation studies to explore the effects of specific parameter configurations (i.e., combinations of high vs. low drift rate and boundary separation parameters) on both the diagnostic accuracy and parameter recovery.

## Follow-up simulations

We conducted a follow-up simulation study in which we generated 1000 datasets with 160 participants ($P = 160$), 40 trials per condition ($T = 40$), using the same four effect size levels as in our extended simulation study ($\beta \in \{0.0, 0.1, 0.2, 0.4\}$). These simulations introduce four distinct true parameter configurations defined by combining two levels of the population intercept of the drift rate (high $\mu _\nu$ vs low $\mu _\nu$) and two levels of the population mean boundary separation (high $\mu _\alpha$ vs low $\mu _\alpha$). These parameter configurations were created by sampling true parameter values from different uniform distributions, as indicated below:

$$\begin{aligned} \mu _{\nu }^{\textrm{low}}&\sim \mathcal {U}\!\left( 0, 1\right)&\mu _{\nu }^{\textrm{high}}&\sim \mathcal {U}\!\left( 2, 3\right) \nonumber \\ \mu _{\alpha }^{\textrm{low}}&\sim \mathcal {U}\!\left( 2, 2.5\right)&\mu _{\alpha }^{\textrm{high}}&\sim \mathcal {U}\!\left( 3.5, 4\right) \nonumber \end{aligned}$$

The population mean non-decision time ($\mu _\tau$) and all individual-level parameters were generated in the same way as in our first simulation study.

**Fig. 4:**
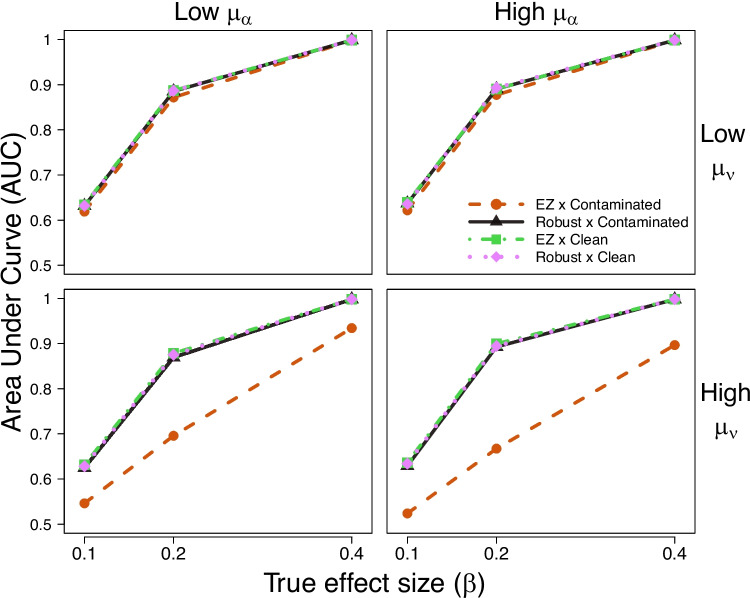
Diagnostic performance of the robust and standard implementations of the EZ-DDM under clean and contaminated data across different parameter configurations. Each panel shows the AUC (*vertical axis*) for different true effect sizes (*horizontal axis*) and simulation comparison conditions (different lines). The *left panels* show the results for low levels of the population boundary separation parameter ($2 \le \mu _\alpha \le 2.5$) and the *right panels* show the results for high levels of the population boundary separation parameter ($3.5 \le \mu _\alpha \le 4$). The *top panels* correspond to low levels of the population intercept drift rate parameter ($0 \le \mu _\nu \le 1$) and the *bottom panels* correspond to high levels of the population intercept drift rate parameter ($2 \le \mu _\nu \le 3$)

### Diagnostic accuracy by parameter configuration

We evaluated the diagnostic performance of the standard and robust implementations of the EZ-DDM under clean and contaminated data across parameter configurations. Results are summarized in Fig. 4, where the two levels (high vs. low) of the population intercept for the drift rate parameter $\mu _\nu$ and the two levels (high vs. low) of the population mean boundary separation parameter $\mu _\alpha$ are organized across rows and columns, respectively. Just like in Fig. 1, each panel in Fig. 4 shows the AUC (vertical axis) as a function of the true effect size (horizontal axis) across all simulation comparison conditions (different lines). The main result highlighted by Fig. 4 is that both implementations of the EZ-DDM perform similarly across data conditions when the true population intercept for the drift rate is low (i.e., $0 \le \mu _\nu \le 1$, as it would be in a hard task condition). However, when the population intercept is high (i.e., $2 \le \mu _\nu \le 3$, as it would be in an easy task condition), the standard implementation shows a notable decrease in AUC performance under contaminated data. This result holds across low and high levels of the population boundary separation parameter ($2 \le \mu _\alpha \le 2.5$ and $3.5 \le \mu _\alpha \le 4$).

**Fig. 5:**
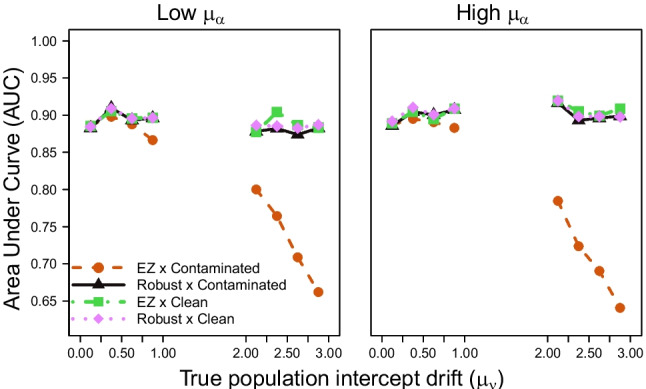
AUC plotted as a function of the true population intercept for the drift rate parameter ($\mu _{\nu }$) in simulations with fixed effect size $\beta = 0.2$ and a within-subject *t* test design on the drift rate parameter with 40 trials per condition across 160 participants. Population intercept drift rate values are aggregated into bins and shown on the *horizontal axis*, with the *vertical axis* showing the AUC. Panels distinguish between true population boundary separation ($\mu _\alpha$) conditions, with low boundary separation values shown on the left and high boundary separation values shown on the right. We use different lines to distinguish between the simulation comparison conditions

**Fig. 6:**
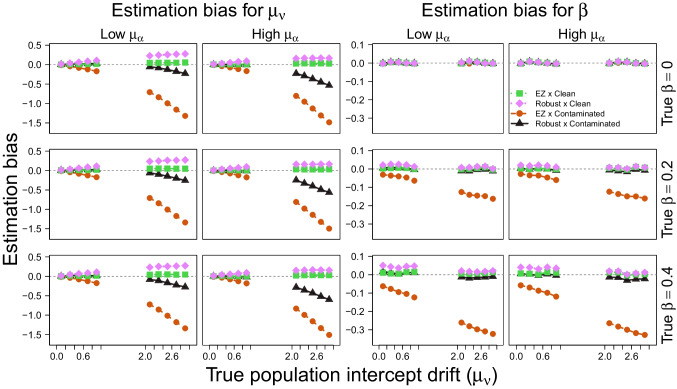
Estimation bias for the population intercept drift rate $\mu _\nu$ (*first two columns*) and the effect size parameter $\beta$ (*last two columns*) as a function of the true population intercept drift rate $\mu _\nu$ (*horizontal axis*) across high and low levels of the population boundary separation parameter $\mu _\alpha$ (first and third columns vs. second and fourth columns, respectively), and different levels of the true effect size $\beta$ (different rows). All simulation comparison conditions are shown in different lines

We further inspect these results by examining changes in the AUC as a function of the true population intercept for the drift rate parameter $\mu _\nu$, across high and low levels of the population boundary separation parameter $\mu _\alpha$, for a fixed effect size $\beta = 0.2$ (which was the value for which we observed the largest vertical distance in Fig. 4). Results are summarized in Fig. 5, where the AUC is plotted as a function of the true population intercept for the drift rate parameter $\mu _\nu$ (aggregated across ten bins: five “low” and five “high” bins), with panels corresponding to low (left column) and high (right column) values of $\mu _\alpha$. This figure confirms that the AUC for the standard implementation of the EZ-DDM under contaminated data decreases as the true population intercept for the drift rate parameter $\mu _\nu$ increases, across both levels of the population boundary separation parameter $\mu _\alpha$.

### Estimation performance by parameter configuration

To further understand the differences in AUC observed for the standard EZ-DDM under contaminated data, we examined how different true values of the population intercept for the drift rate parameter $\mu _\nu$ affect the estimation of both the effect size parameter $\beta$ and $\mu _\nu$ itself. Results are summarized in Fig. 6, where the first two columns show the estimation bias for $\mu _\nu$ and the last two columns show the estimation bias for $\beta$. The first and third columns correspond to low values of the boundary separation parameter $\mu _\alpha$, whereas the second and fourth columns correspond to high values of $\mu _\alpha$. Each row corresponds to a different true effect size $\beta$ level. Within each panel, the vertical axis shows the estimation bias (computed as the mean difference between the true and the estimated values based on posterior means), and the horizontal axis aggregates values of the true population intercept for the drift rate parameter $\mu _\nu$ into ten bins.

Figure 6 shows that under contaminated data, the population intercept for the drift rate parameter $\mu _\nu$ is increasingly underestimated as its true value increases across both implementations of the EZ-DDM, with the robust implementation showing less severe underestimation. As expected, the underestimation appears to be independent of the effect size parameter $\beta$. However, the underestimation is more pronounced when the population boundary separation parameter $\mu _\alpha$ is high. On the other hand, Fig. 6 also shows that, under contaminated data, the standard implementation of the EZ-DDM underestimates the effect size parameter $\beta$ towards 0, with the magnitude of underestimation increasing as its true value increases and near-zero bias for $\beta = 0$.

Taken together, these results provide an intuitive account of the AUC differences observed in Fig. 4. The AUC reflects how well the model can discriminate between null and true effects, which, given our simulation study design, corresponds to detecting differences in drift rate across conditions within participants. When the population intercept for the drift rate parameter $\mu _\nu$ is high, contamination induces a systematic underestimation of both $\mu _\nu$ and $\beta$ in the standard implementation of the EZ-DDM, attenuating the estimated within-subject difference in drift rate between conditions. As a result, the estimated effect sizes $\beta > 0$ are pulled closer to zero, reducing their separation from the null condition ($\beta = 0$). This attenuation makes it more difficult to distinguish datasets generated under the null from those generated under true effects, increasing the likelihood of type II errors (i.e., failing to detect true effects when they are present) and bringing AUC values closer to 0.5. In contrast, the robust implementation mitigates this attenuation by reducing the bias in both parameters, which preserves the within-subject differences in drift rate induced by $\beta$, allowing the robust implementation to maintain its diagnostic accuracy under contamination.

## Discussion and conclusion

The motivation for this study was to address concerns about the sensitivity of the EZ-DDM framework to contaminant data, which limits its applicability to real-world data sets. To this end, we proposed a robust implementation of the EZ-DDM in which the mean and variance of the observed RTs are replaced by the median and a robust variance estimator. The performance of this robust variant was evaluated in an elaborate simulation study using a factorial design that crossed data integrity (cleaned vs. contaminated) and summary method (mean/variance vs. quantile-based), across varying levels of sample size, trial count, and true effect size.

The results from the simulation study show that the robust version performed indistinguishably from the standard EZ-DDM when applied to clean data. Crucially, the robust implementation remained consistent under contamination conditions that otherwise substantially hindered the performance of the EZ-DDM. This general pattern was observed both in terms of the diagnostic accuracy (AUC) and parameter recovery performance (RMSE and estimation bias of the estimated effect size parameter $\beta$). Overall, these results suggest that the robust replacement for the standard EZ-DDM maintains its efficiency even under data contamination.

We further investigated the differences in performance found between both implementations across data integrity conditions through a series of follow-up simulations in which we controlled for the values of the population drift rate and boundary separation parameters. This inspection revealed that the diagnostic accuracy of the standard implementation under contaminated data decayed as a function of the true population intercept drift rate across both high and low levels of the population boundary separation parameter. This pattern could also be found in the parameter recovery performance of both the effect size parameter and the population intercept drift rate parameter, which were increasingly underestimated as the true population intercept drift rate increased (with the effect size parameter showing larger biases for larger true effect size values). Altogether, these results suggest that the standard implementation of the EZ-DDM is most affected by the presence of contaminant data when the drift rate is high, such as when the task difficulty is low, and where we expect to find lower RTs, and thus, the presence of outliers has a more pronounced impact on the standard mean and variance summary statistics.

To conclude, the robust implementation of EZ-DDM provides a simple and efficient alternative to the standard EZ-DDM, combining computational tractability with resilience to contamination. Under all conditions, the robust version performed as well or better than the original implementation of EZ-DDM. We recommend that researchers use the robust EZ-DDM in all cases.
